# Independent Colimitation for Carbon Dioxide and Inorganic Phosphorus

**DOI:** 10.1371/journal.pone.0028219

**Published:** 2011-12-01

**Authors:** Elly Spijkerman, Francisco de Castro, Ursula Gaedke

**Affiliations:** Department of Ecology and Ecosystem Modelling, University of Potsdam, Potsdam, Germany; Mt. Alison University, Canada

## Abstract

Simultaneous limitation of plant growth by two or more nutrients is increasingly acknowledged as a common phenomenon in nature, but its cellular mechanisms are far from understood. We investigated the uptake kinetics of CO_2_ and phosphorus of the algae *Chlamydomonas acidophila* in response to growth at limiting conditions of CO_2_ and phosphorus. In addition, we fitted the data to four different Monod-type models: one assuming Liebigs Law of the minimum, one assuming that the affinity for the uptake of one nutrient is not influenced by the supply of the other (independent colimitation) and two where the uptake affinity for one nutrient depends on the supply of the other (dependent colimitation). In addition we asked whether the physiological response under colimitation differs from that under single nutrient limitation.

We found no negative correlation between the affinities for uptake of the two nutrients, thereby rejecting a dependent colimitation. Kinetic data were supported by a better model fit assuming independent uptake of colimiting nutrients than when assuming Liebigs Law of the minimum or a dependent colimitation. Results show that cell nutrient homeostasis regulated nutrient acquisition which resulted in a trade-off in the maximum uptake rates of CO_2_ and phosphorus, possibly driven by space limitation on the cell membrane for porters for the different nutrients. Hence, the response to colimitation deviated from that to a single nutrient limitation. In conclusion, responses to single nutrient limitation cannot be extrapolated to situations where multiple nutrients are limiting, which calls for colimitation experiments and models to properly predict growth responses to a changing natural environment. These deviations from single nutrient limitation response under colimiting conditions and independent colimitation may also hold for other nutrients in algae and in higher plants.

## Introduction

Plant biomass forms the basis of food webs and its primary production promotes global economic and ecosystem services such as crop harvest, fish yield and carbon sequestration. Because plant photosynthesis and growth is often nutrient limited, knowledge on how nutrients limit plant growth is both, ecologically and economically important. On the other hand, massive plant growth such as large scale algal blooms, arising from an excess of nutrients, negatively affect biodiversity and are a nuisance to human activity. This excessive plant growth often results from plants acclimated to scavenge the limiting nutrient but which are suddenly faced with saturating conditions often due to antropogenic impacts. Thus, knowledge on plant nutrient uptake kinetics and response to changes of the limiting nutrient provides important insights to predict plant growth response.

Most previous studies have focused on the effect of a single limiting nutrient, such as inorganic phosphate (P) which often limits algal growth in freshwater (e.g. [1]). In many cases, however, this approach was unsatisfactory, which has recently been explained by the occurrence of colimitation by two or more nutrients under natural conditions [2], [3]. For example, a colimitation by nitrogen, P and iron was shown in the phytoplankton communities of Lake Kasumigaura [4] and Lake Erie [2]. As illustrated in these two studies, nutrient supplementation alleviates each incremental limitation and produces a synergistic effect when all limiting nutrients are added together (see [5] for a nice illustration). In the case of entire plankton communities a stepwise increase in growth and biomass after addition of all limiting nutrients, possibly results from the independent response of different species. In addition, a single species can also show the effects of colimitation [6], [7].

**Figure 1:**
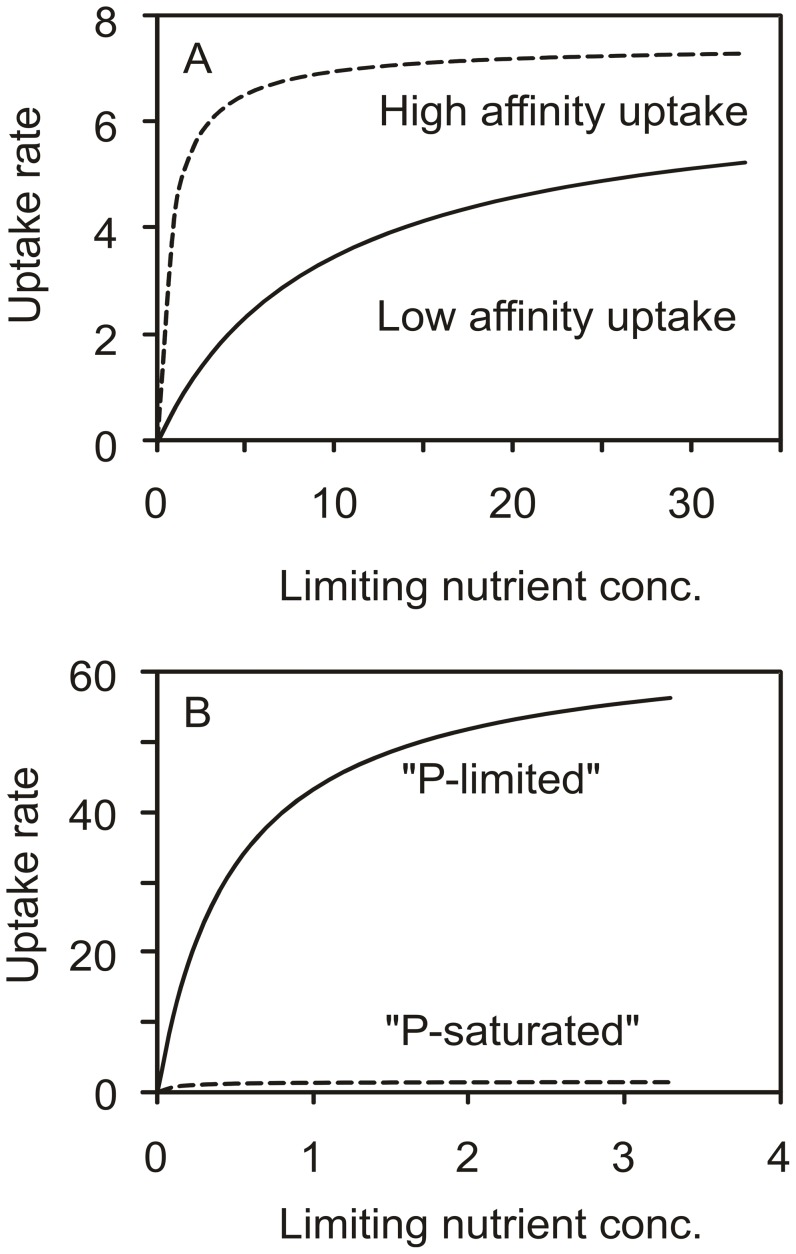
Illustration of P uptake kinetics: Cell-based rates related to the initial P-concentration in the medium (µM P). A) Data from [61] on the high (K_m_ 0.7 µM P) and low (K_m_ 9.2 µM P) affinity P transporter in *Escherichia coli* that have similar V_max_, B) Data from [31] on P-limited (high V_max_) and P-saturated (low V_max_) *Chlamydomonas acidophila* with the same K_m_ (0.1–0.5 µM P).

In general, algal cells respond to nutrient limiting conditions by increasing their ability for nutrient uptake. This can be achieved in two ways which are not mutually exclusive (Fig. 1); either increasing the maximum uptake rate (V_max_) and/or increasing the affinity for uptake. The latter is typically reflected in a decrease of the half saturation constant (K_m_), and an increase of the initial slope of the curve (affinity characterized by V_max_:K_m_). V_max_ is positively related to the *number* of porters in the cytoplasmic membrane [8] whereas changes in K_m_ reflect different *types* of porters. The affinity thus reflects the physiological combination of the two strategies of acclimation.

In response to a P-limitation most algae increased V_max_ as found in the green alga *Scenedesmus* sp. [9], the diatom *Thalassiosira pseudonana* [10] and the cyanobacterium *Anabaena flos-aquae* [11], while the K_m_ often remained relatively constant. In contrast, in response to low CO_2_ many algae decrease their K_m_ [12]–[14]. Both responses often result in an enhanced affinity for nutrient uptake; likely driven by the need to maintain a balanced cell nutrient content. Cell homeostasis is of great importance for a proper functioning of enzymes and proteins, and consequently nutrient uptake and excretion rates are regulated to balance nutrient ratios within a certain range [15], [16].

Even if colimitation is a rather common phenomenon in phytoplankton and plant growth in general [3], [5], the cellular response mechanisms in a single algal species are far from understood. They may involve interactions between two or more nutrients in short supply which questions the common practice to infer the consequences of nutrient limitation by considering only a single nutrient at a time. Consequently, we ask whether a colimitation by two macronutrients alters their respective uptake kinetics, compared to situations where a single nutrient is limiting, to better understand the mechanism(s) of algal growth acclimation and cellular response to colimiting conditions.

Here, we study the uptake and growth of the single-celled flagellated green alga *Chlamydomonas acidophila* to limiting conditions of CO_2_ and P, two important macro-nutrients for phytoplankton which are prone to change in the future and often limit phytoplankton blooms in very acidic and neutral fresh waters, as well as in marine systems [17]–[20]. Inorganic carbon occurs in three forms in aquatic systems: CO_2_, HCO_3_^−^ and CO_3_^2−^, but here we consider only the dissolved gas, CO_2_, which is the only inorganic C source present in the low pH environment (pH 2.7) of our model organism [21]. In *C. acidophila*, CO_2_ uptake is considered an active process supported by a high affinity uptake mechanism at least under low CO_2_ conditions [12], thus allowing us to consider CO_2_ as a ‘normal’ macro-nutrient.

Recent theoretical studies suggest two types of colimitation for macro-nutrients: 1) independent and 2) dependent colimitation [6], [7], [22]. An independent colimitation arises when the concentration of more than one nutrient is below the optimal concentration for uptake (a multi-nutrient colimitation sensu [6]). Under these conditions, the cell will increase V_max_ and/or decrease the K_m_ for the uptake of both limiting nutrients, i.e. exhibit a multiplicative response to the two concurrent limitations. The response to an independent colimitation might be restricted according to theoretical considerations showing that V_max_ is positively related to the number of nutrient transporter proteins [8] and as space on the cell surface for these proteins may be limited [8], [23], the total number of nutrient transporters is thus restricted. Hence, theory predicts a trade-off between the V_max_ of both limiting nutrients on a cellular level depending on the limiting nutrient in highest demand [24]. However, as far as we know, this hypothesis has never been tested with empirical data.

Alternatively, a dependent colimitation for nutrients exists if the uptake of one nutrient is enhanced by the availability of another one (a biochemical colimitation sensu [6]). In our situation, a P-limitation may inhibit the high affinity uptake of inorganic carbon (i.e. prevent a low affinity constant for CO_2_ uptake (K_m,C_)) and of a CO_2_ concentrating mechanism (CCM, [25]–[27]) which are both active processes [26], [28] that depend, directly or indirectly, on ATP. Under P-saturated and low CO_2_ conditions, *C. acidophila* had a low K_m,C_ and a CCM, both of which were absent under high CO_2_ conditions [12], [29] indicating some costs which promote their down regulation at sufficient C-supply. If CO_2_ uptake depends on P-supply, the K_m_ for CO_2_ uptake should increase with increasing P-limitation (as shown in the green alga *Chlorella emersonii* [25]). The dependent colimitation hypothesis implies that a minimum P concentration is required to acclimate to low CO_2_. If that minimum is not satisfied it results in a high K_m,C_ when both P and CO_2_ are low. The alternative, that P uptake ability depends on CO_2_-concentration, is also possible. Recent studies on *C. acidophila* [30] support this option: at high CO_2_ concentrations, cells could deplete the P concentration in the medium more strongly than at low CO_2_ concentrations. Also, the minimum cellular P quota was lower in high than in low CO_2_ cells [31], suggesting that cellular P requirements for growth are lower at high CO_2_. Following this hypothesis, the P uptake ability (enhanced maximum P uptake rate (V_max,P_) and/or decreased affinity constant for P uptake (K_m,P_)) should increase with increasing CO_2_-concentration.

We analyzed the response of *C. acidophila* to a combination of CO_2_ and P limitations. Our objectives are first, to test the hypothesis that responses to two limiting factors cannot be inferred from single-nutrient studies; second to decide which of the above-described mechanisms of colimitation is more plausible. For this, four different models, two of independent colimitation and two of dependent colimitation (CO_2_ limiting P uptake and *vice versa*), were fitted to the data by maximum likelihood. We also provide empirical evidence for the theoretically expected trade-off between the V_max_ for both nutrients.

## Methods

### Cultures and analyses

Triplicate semi-continuous cultures of *C. acidophila* Negoro (CCAP 11/137) were grown at 20±1°C in Woods Hole medium [32] with 1.6 µM P and a pH adjusted to 2.7 with HCl. Daily diluted cultures at growth rates of 0.1, 0.2, 0.3, 0.4 and 0.6 d^−1^ in low CO_2_ and 0.1, 0.2, 0.4, 0.6 and 0.8 d^−1^ in high CO_2_ treatments resulted in a decrease of P-limitation with increasing growth rate. Cell densities ranged between 1.1 10^5^ cells ml^−1^ in the highest growth rates to 1.5 10^6^ cells ml^−1^ in the lowest growth rates. Cell densities were on average 1.6-fold higher in the high CO_2_ cultures than in the low CO_2_. Incident light was approximately 200 µmol photons m^−2^ s^−1^ in all cultures with a light/dark period of 16/8 h. Daily dilution and harvesting were done 4–5 hours after the onset of light. High CO_2_ cultures were mildly aerated with a mixture of 4.5% CO_2_ in air, resulting in an average CO_2_ concentration in the medium of 0.33 (±0.05, n = 20) mM C, whereas low CO_2_ cultures were non-aerated to realise CO_2_ limiting conditions and contained approximately 0.02 mM CO_2_ (HighToc, Elementar, Hanau, Germany). These concentrations were measured in the medium, but do not reflect concentrations nearby the cell (see discussion for further details). Inorganic iron buffered the pH of the medium, thus resulting in a constant pH independent of CO_2_ concentration. At balanced growth (remaining at constant cell density 4–5 hours after the onset of light after an exchange of three to five times the culture volume), samples were taken for measurements of algal density, chemical analyses and CO_2_- and P-uptake kinetics.

Cellular phosphorus quota (Q_p_), cellular C and P content were determined by measuring the particulate P and C in the cultures. The particulate P concentration was determined on filtered culture suspension (0.2 µm Whatman nucleopore) extracted at 100°C for 1 h with K_2_S_2_O_8_ and 0.5 M H_2_SO_4_ and measured spectrophotometrically using molybdate and ascorbic acid [33]. Particulate C was analysed on culture suspension filtered on pre-combusted GF/F filters (Whatman), dried for one week at 50°C, and combusted in a carbon analyser (HighTOC+N, Elementar or EuroVector CHNS-O Elementaranalysator, Wegberg, Germany). Cell numbers were determined using an automatic cell counter (CASY 1, Model TT, Schärfe, Reutlingen, Germany).

### Uptake kinetics

The CO_2_-uptake kinetics was obtained in a temperature regulated light dispensation system (Topgallant LLC, Salt Lake City, Utah, USA) providing 500 µmol photons m^−2^ s^−1^ and measuring oxygen evolution in a Clark type electrode (Micro-electrode Inc., Bedford, Ohio, USA) as described for P-replete *C. acidophila* in [12], [29]. Cells were centrifuged (1500*g*, 5 min) and resuspended in C-free medium to an optical density of 0.2 at 750 nm in a 1 cm cuvette. After O_2_ evolution ceased, one of six different concentrations of HCO_3_^−^ was added and the response recorded on a computer. Each concentration was measured in three-fold, resulting in 18 data points for establishing one kinetic curve. At pH 2.65, 95% of the added HCO_3_^−^ was assumed to be dehydrated to CO_2_ within 60 s and no delay in response in O_2_ evolution was observed. There was no significant effect of the addition of HCO_3_^−^ on the pH: on average pH decreased by 0.001 units over the total run of 6 additions. Part of the algal suspension was fixed with iodine and cell densities were determined as described above. Oxygen evolution rates were related to cell densities and fitted to the Michaelis Menten model using the non-linear regression module in SPSS software (using the Levenberg Marquardt estimation, version 12.01) to obtain the K_m,C_ and the maximum CO_2_ uptake rate by photosynthesis (V_max,C_).

For P-uptake kinetics, cells were centrifuged (1500*g*, 5 min) and the pellet resuspended in medium without P and iron-EDTA at a pH of 2.7. Final densities were adjusted to an optical density of 0.02 at 750 nm in a 1 cm cuvette. After an acclimation of 15 to 30 min at 90 µmol photons m^−2^ s^−1^, P-uptake was determined over a period of one minute after the addition of different concentrations of H_3_^33^PO_4_ (111 TBq mmol^−1^ specific activity, Amersham biosciences, Freiburg, Germany) diluted in stock solutions of 50 or 500 µM K_2_HPO_4_ at pH 2.7 as described in [31]. Uptake was terminated by filtration on 1.2 µm cellulose acetate filters and subsequently rinsed with 0.2 M LiCl. P-uptake kinetics from two out of three replicate P-limited cultures and data of P-replete cells were published before [31]. Similar as for CO_2_-uptake kinetics, cell specific data were fitted to the Michaelis-Menten model to estimate the K_m,P_ and V_max,P_.

### Modelling and statistics

We fitted four models describing colimitation to our data, following terminology and equations from [22] and [7]. This was done by calculating the external P and CO_2_ (C) concentrations from individual uptake kinetics and cellular quota for each balanced growth rate following principle characteristics of the (semi-) continuous culture as explained by several authors [31], [34]–[37]. Direct measurements of the limiting nutrient concentrations in the medium were not possible as they were too low to be measured directly, but the calculation of external concentration is a reasonable procedure despite the inevitable uncertainty involved [36]. Prior to this, we tested single nutrient models with the standard Monod function for P and C. In both cases the fit was poorer than in two-nutrient (colimitation) models, so we do not show detailed results here (Fig. S1). We used the following models describing colimitation:

1a. Independent co-limitation, multiplicative form:
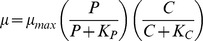
1b. Independent co-limitation, minimum form (Liebig's Law):
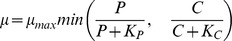
2a. Dependent co-limitation with C acquisition depending on P-limitation:
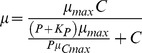
where the first term of the denominator comes from:


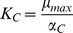
 and since: 
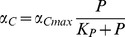
 it follows that: 
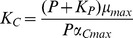


2b. Dependent co-limitation with P acquisition depending on C-limitation:
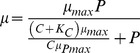



The symbols used in the equations are *μ*, growth rate (in h^−1^); *μ*_max_, maximum growth rate (in h^−1^); *α*_C_, *α*_P_, affinity for growth at C or P-limiting conditions; *α*_Cmax_, *α*_Pmax_, maximum affinity for growth at C or P-limiting conditions; *K_C_*, half saturation constant for growth in relation to external CO_2_ concentration and *K_P_*, half saturation constant for growth in relation to external P concentration (for details see [7], [22]). We used maximum likelihood to fit the models to the data, assuming a normal distribution for the stochastic component of the models.

In addition to the Monod curves we established contour plots to distinguish between the effects of the cellular C and P contents on the uptake kinetics using Matlab 7.8 and interpolation based on Sandwell [38].

Statistical tests were performed with SPSS (version 12.01). Homogeneity of variances was checked with a Levene test.

## Results

We tested the nutrient uptake response in the green alga *Chlamydomonas acidophila* to different CO_2_ and P colimiting conditions. By using semi-continuous cultures the extent of limitation decreases with increasing dilution rate and, thus, with increasing balanced growth rate.

**Figure 2:**
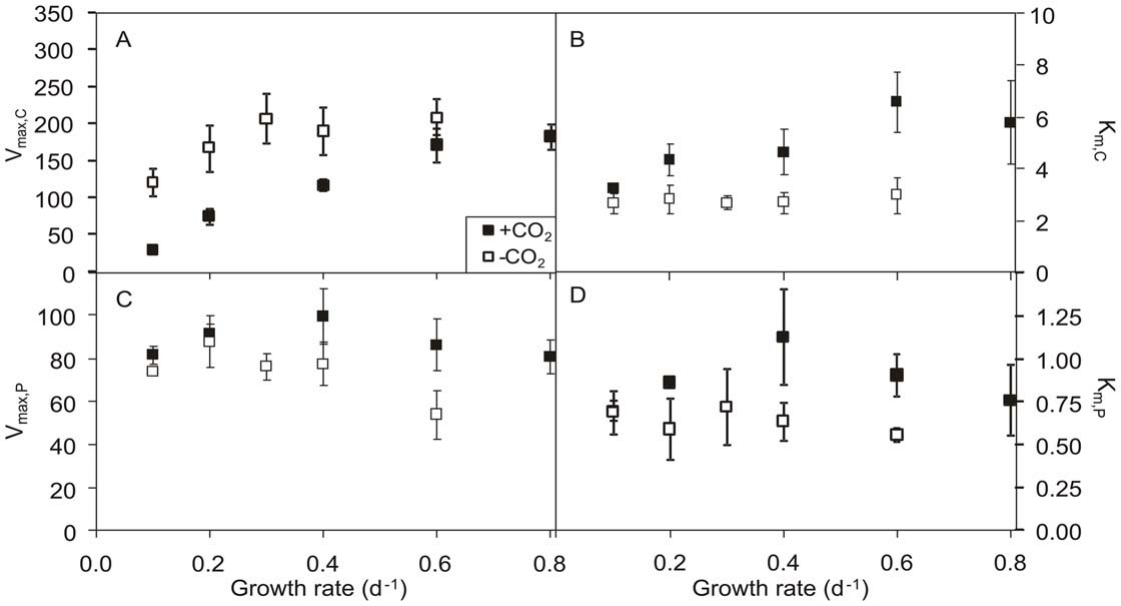
CO_2_ and P uptake kinetics of *Chlamydomonas acidophila* in relation to balanced growth rates at high CO_2_ (+CO_2_) and low CO_2_ (−CO_2_) P-limited conditions. A) Maximum CO_2_ uptake rate by photosynthesis (V_max,C_, mmol O_2_ 10^−12^ cells h^−1^), B) affinity constant for CO_2_ uptake by photosynthesis (K_m,C_, µM CO_2_), C) maximum P uptake rate (V_max,P_, mmol P 10^−12^ cells h^−1^) and, D) affinity constant for P uptake (K_m,P_, µM P). Mean ± SE of 3 replicates.

CO_2_ and P uptake kinetics differed in the high and low CO_2_ acclimated algae (Fig. 2), e.g. V_max,C_ was higher in the low CO_2_ acclimated cultures than in the high CO_2_ ones, when the effect of growth rate was accounted for (Fig. 2A; ANCOVA, F_1,27_ = 27.0, p<0.001). Possibly, this kinetic difference resulted from the lower cellular C content (Fig. S2A) in the low CO_2_ than in the high CO_2_ cells at a given steady state growth rate. The higher V_max,C_ in cells with low C content supports the nutrient kinetic response theory (Fig. 1B) that at a cellular level, a C-deficiency results in a higher CO_2_ demand and thus a higher V_max,C_. The potential growth rate (growth capacity) calculated from V_max,C_ and the cellular carbon content supports the idea that conditions were limiting for CO_2_, as the growth capacity was between 1.7 and 4.4-fold higher than balanced growth rates in the low but not in the high CO_2_ cultures (Table S1). The ratio between growth capacity and balanced growth rate increased with decreasing growth rate, i.e. with increasing CO_2_ limitation (see also [39]). In high CO_2_ cells the growth capacity equaled the balanced growth rate, but enrichment experiments showed that these cells were nevertheless colimited for P and CO_2_ [39] meaning that results from C-uptake kinetics alone were not conclusive.

In low CO_2_, V_max,C_ was the same in P-limited cells (data from the three highest growth rates in Fig. 2) and P-replete (data from [12]), whereas in high CO_2_ it was lower in P-limited than in P-replete cells (Table 1). These results illustrate that the kinetic response to a colimitation differs from that to a single nutrient limitation. In contrast, the K_m,C_ was the same in P-limited and P-replete cultures (Table 1), but depended on the CO_2_ concentration: K_m,C_ was higher in the high CO_2_ than in the low CO_2_ cultures (ANCOVA, F_1, 27_ = 10.5, p<0.01).

**Table 1:** CO_2_ and P uptake kinetics of *C. acidophila* in P-replete batch cultures (data from [12], [31]) and in P-limited cultures (data from the three highest growth rates in Fig. 2 in this contribution).

	P-replete	P-limited	Statistical result
Low CO_2_ V_max,C_:	178±20	202±14	ANOVA, F = 0.86, df = 1,12, p = 0.37
High CO_2_ V_max,C_:	311±16	161±13	ANOVA, F = 47.1, df = 1,17, p<0.001
Low CO_2_ K_m,C_:	2.4±0.3	3.1±0.4	ANOVA, F = 1.5, df = 1,12, p = 0.24
High CO_2_ K_m,C_:	5.7±0.5	5.7±0.6	
Low CO_2_ V_max,P_	3±1	69±9	ANOVA, F = 23.9, df = 1,9, p<0.001
Low CO_2_ K_m,P_	0.23±0.10	0.64±0.07	ANOVA, F = 5.6, df = 1,9, p<0.05

Values of V_max,C_ given in mmol O_2_ 10^−12^ cells h^−1^, K_m,C_ in µM CO_2_, V_max,P_ in mmol P 10^−12^ cells h^−1^ and K_m,P_ in µM P. Mean ± SE of at least 3 measurements.

Contrary to expectations, K_m,C_ did not increase with decreasing growth rate in the low CO_2_ cultures (Fig. 2B; Pearson r_15_ = 0.40, p = 0.14), which should happen if CO_2_ uptake depended on P-limitation (model 2a). Moreover, K_m,C_ decreased with decreasing growth rate in the high CO_2_ cultures (Fig. 2B; Pearson r_15_ = 0.55, p<0.05), thus suggesting that the high CO_2_ cells became more severely CO_2_-limited with lower growth rates (see discussion for a possible mechanism). Although at such low growth rates high CO_2_ cells were severely P-limited, a high affinity CO_2_ uptake kinetics was established also suggesting that a P-limitation did not influence CO_2_-acquisition.

Under low CO_2_ conditions V_max,P_ did not vary over growth rate (Fig. 2C), suggesting that all cultures were severely P-limited. This V_max,P_ was 20-fold higher in P-limited than in P-replete cells (data from [31]; Table 1). In the colimited cultures V_max,P_ was on average higher in high CO_2_ than in the low CO_2_ cultures (ANCOVA, F_1, 24_ = 12.4, p<0.01) and coincides with a lower cellular P content in the high CO_2_ cells (Fig. S2B). Thus, high CO_2_ cells could exploit the external P concentration more and were possibly more severely P-limited than low CO_2_ cells resulting in a higher calculated growth capacity (Table S1). Growth P capacities (i.e. the hypothetical growth rate at V_max_) greatly exceeded the balanced growth rate in all cultures. The higher V_max,P_ in the high CO_2_ cultures suggests that P-uptake depended on CO_2_ during growth (possibly supporting model 2b).

K_m,P_ did not vary over growth rate in either high or low CO_2_ treatments, but was higher in the high CO_2_ than in the low CO_2_ cultures (Fig. 2D; T-test, t_27_ = 3.1, p<0.005), suggesting that low CO_2_ cells had a higher affinity P-uptake system. Possibly, V_max,P_ influenced the estimation of this parameter as *C. acidophila* had a high affinity P-uptake system under all conditions, including P-replete conditions (Table 1). Because the cellular C and P content of the high and low CO_2_-acclimated cells differed at a given balanced growth rate (Fig. S2) and we expected cell homeostasis to play a role, we will now relate the uptake kinetics to the cellular P to C quota which is independent of cell size.

**Figure 3:**
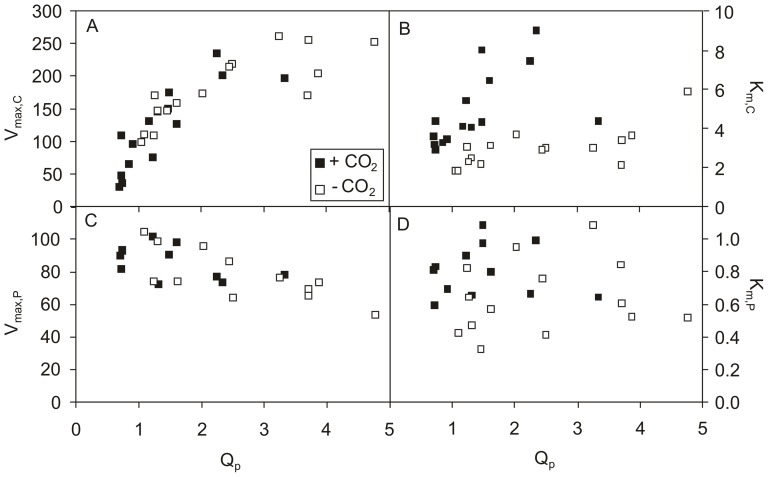
CO_2_ and P uptake kinetics of *Chlamydomonas acidophila* in relation to the cellular P quota (Q_p_, mmol P mol C^−1^) grown in high CO_2_ (+CO_2_) and low CO_2_ (−CO_2_) P-limited conditions. A) Maximum CO_2_ uptake rate (V_max,C_, mmol O_2_ 10^−12^ cells h^−1^), B) affinity constant for CO_2_ uptake by photosynthesis (K_m,C_, µM CO_2_), C) maximum P uptake rate (V_max,P_, mmol P 10^−12^ cells h^−1^) and, D) affinity constant for P uptake (K_m,P_, µM P).

**Figure 4:**
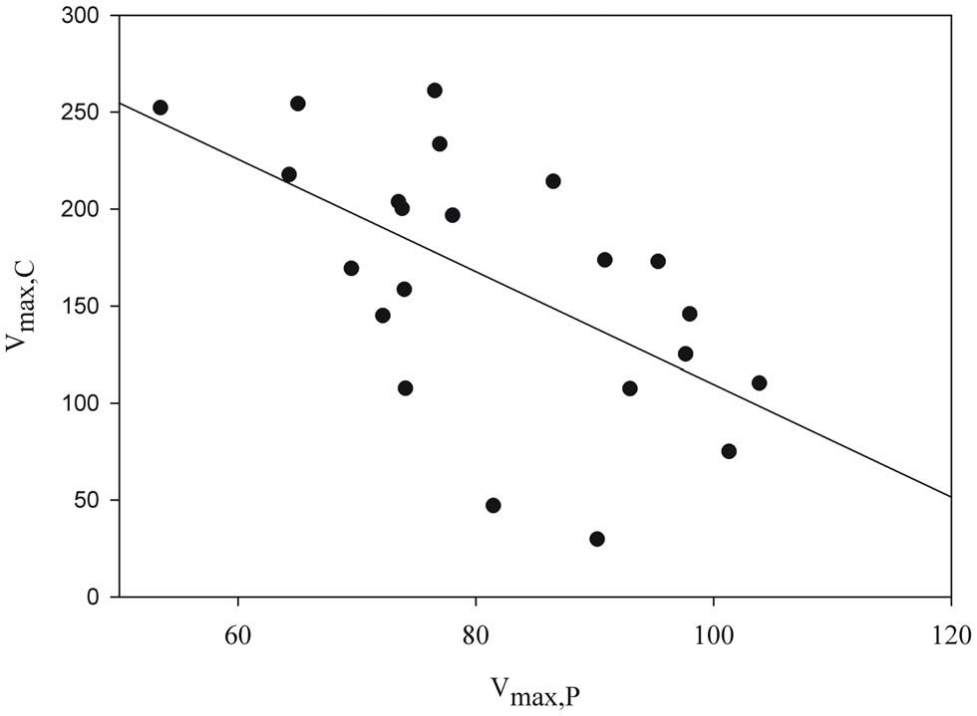
Maximum CO_2_ uptake rate (V_max,C_ in mmol O_2_ 10^−12^ cells h^−1^) in relation to the maximum P uptake rate (V_max,P_ in mmol P 10^−12^ cells h^−1^) of the same colimited culture of *Chlamydomonas acidophila*.

**Figure 5:**
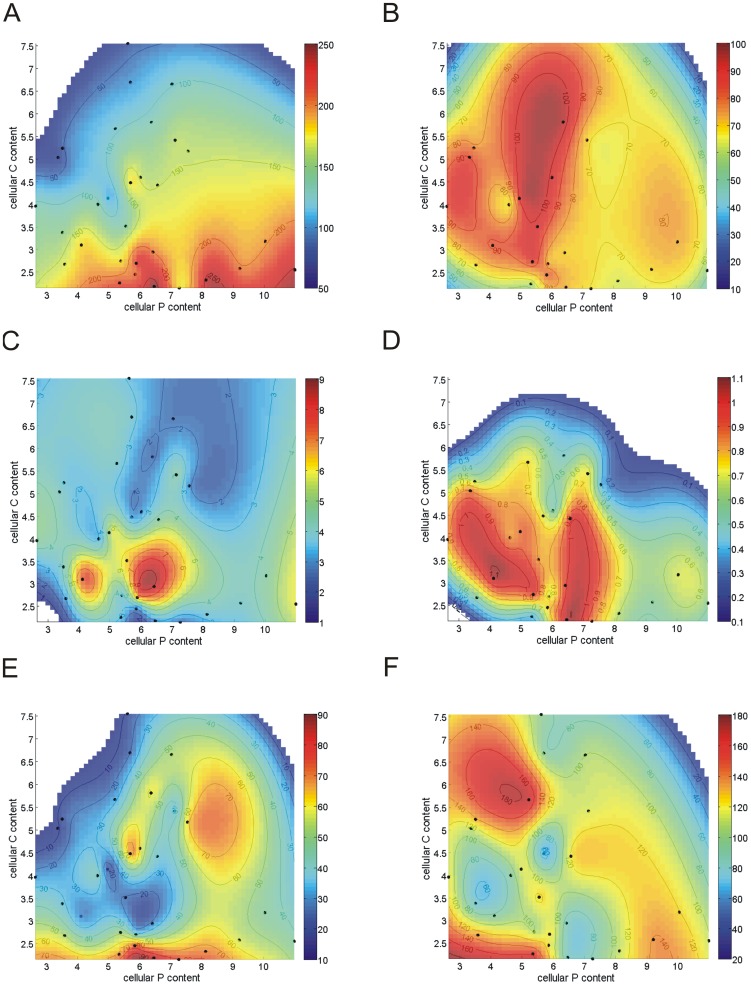
Contour plots of individual measurements of CO_2_ and P uptake kinetics in relation to the cellular C (in pmol C cell^−1^) and cellular P (in fmol P cell^−1^) content of *Chlamydomonas acidophila* grown in CO_2_/P-colimited cultures. A) maximum CO_2_ uptake rate (V_max,C_, mmol O_2_ 10^−12^ cells h^−1^), B) maximum P uptake rate (V_max,P_, mmol P 10^−12^ cells h^−1^), C) affinity constant for CO_2_ uptake by photosynthesis (K_m,C_, µM CO_2_), D) affinity constant for P uptake (K_m,P_, µM P), E) affinity for C uptake (V_max,C_:K_m,C_); and F) affinity for P uptake (V_max,P_:K_m,P_). In some parts of the graph, the absence of measured data leads the interpolation algorithm to produce negative values. These values are not plotted.

Independent of CO_2_ conditions the Q_p_ determined V_max_ of both nutrients. V_max,C_ increased (Pearson r_30_ = 0.83, p<0.001) and V_max,P_ decreased with increasing Q_p_ (r_22_ = −0.69, p<0.001; Fig. 3A, C). Consequently, there was a clear trade-off in the V_max_ for both nutrients (Pearson r _22_ = −0.59, p<0.005; Fig. 4). The variation in V_max,C_ was much larger than that in V_max,P_ hence, a small increase of V_max,P_ can only be obtained by a substantial lowering of V_max,C_, implying high costs involved in this adaptation (P-starvation). Cells relatively rich in P (high Q_p_) had a lower V_max,P_, and cells relatively rich in C (low Q_p_) had a much lower V_max,C_. Contour plots which display the cellular C and P content on 2 separate axes, reveal that a low cellular C content resulted in the highest V_max,C_ and a low cellular P content in the highest V_max,P_ (Fig. 5A, B). In addition, there is a tendency for an even higher V_max,C_ at higher cellular P and maximum values of V_max,P_ at higher cellular C contents.

In both high and low CO_2_ conditions, K_m,C_ increased with increasing Q_p_ when data from high and low CO_2_ cultures were analyzed separately (Fig. 3B; Pearson r_15_ = 0.53, p<0.05 for high CO_2_ and Pearson r_15_ = 0.67, p<0.01 for low CO_2_). In addition, K_m,C_ was higher in high than in low CO_2_ cells (Q_p_ as a co-variate; ANCOVA, F_1, 29_ = 25.3, p<0.001). In contrast, no changes in K_m,P_ were observed over Q_p_ (Fig. 3D). The contour plots that separate the cellular C and P content on 2 axes, reveal rather complex patterns of K_m_ in relation to the cellular C and P content (Fig. 5C, D). Against theory (Fig. 1A), cells with a low nutrient content often had a high K_m_ for that nutrient. Observed changes in K_m_ were however small compared to the changes in V_max_, suggesting that overall V_max_ dominated responses in uptake kinetics. As a result, the affinity for C or P uptake was highest at the lowest cellular C and P content, respectively (Fig. 5E, F), although the pattern is less clear than with V_max_ (Fig. 5A, B). That is, the highest affinity for C uptake was realized at the lowest cellular C content with a tendency that the maximum affinity was present in cells with a higher P content (Fig. 5E). The highest affinity for P uptake was realized at the lowest cellular P content but values varied little over cellular C and P content (Fig. 5F).

**Figure 6:**
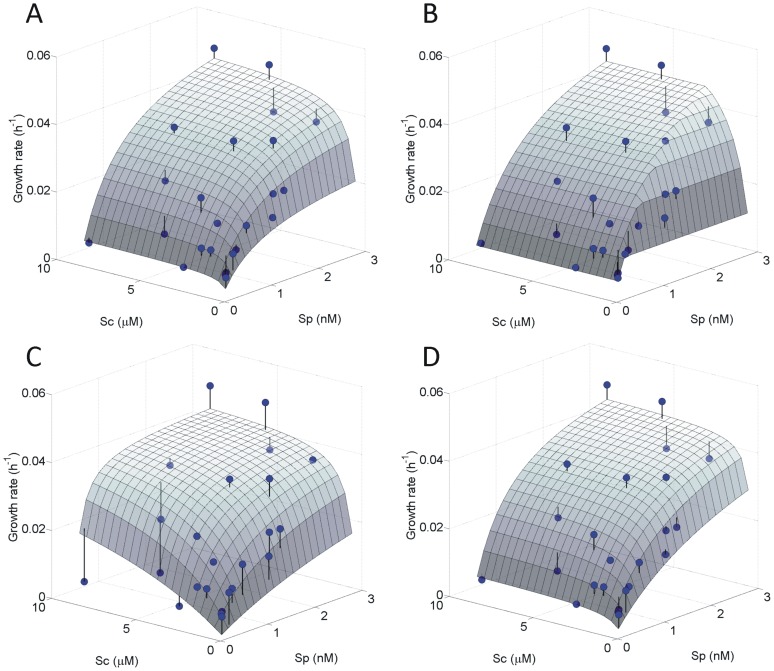
Three-dimensional fit (surface area) of colimitations models to external P and CO_2_ concentrations and the balanced growth rate (h^−1^, red dots). A) model 1a: Independent co-limitation, multiplicative form, B) model 1b: Independent co-limitation, minimum form (Liebig's Law), C) model 2a: Dependent co-limitation (C-uptake depends on P-lim) and, D) model 2b: Dependent co-limitation (P-uptake depends on C-lim). See Table 2 for estimated values of parameters and goodness-of-fit. Please notice the difference in axis between the external CO_2_ (in µM) and P concentration (in nM).

To test for an independent or dependent colimitation, the external P and CO_2_ concentrations were calculated from the kinetic data (concentrations were too low to be measured), thus combining the kinetic characteristics of uptake with the cellular C and P content into a external nutrient concentration present in the medium. Growth rates in relation to these external nutrient concentrations were first fitted to single-nutrient Monod models and revealed that external P concentration could satisfactionally explain growth rate, whereas CO_2_ concentration could not (Fig. S1). Then, we fitted the data to 4 models, reflecting 4 types of colimitation. The best fit of the data was obtained when assuming the multiplicative form of independent colimitation (model 1a; Fig. 6, Table 2) suggesting no interaction between the uptake kinetics of the two limiting nutrients. The fit was better than the single Monod model (Fig. S1), supporting the presence of a colimitation. Figure 6A reveals a strong effect of the external P concentrations on the growth rate, while the effect of the CO_2_ concentration is much weaker. This agrees with the response in the enrichment experiments, where growth was enhanced by P-addition, and CO_2_ addition only stimulated growth when provided in concert with P [39]. Fitting model 1b to the growth rates, which also assumes independent nutrient uptake kinetics but that only the most limiting nutrient (either C *or* P) is affecting the growth rate (ultimately, Liebig's law) resulted in a more angular shape given the sudden changes in nutrient limitation (Fig. 6B; Table 2). The fit of this model to the data was also good, but significantly less than that of model 1A, according to the difference of ∼5 in the Akaike Information Criterion (AIC_c_) between both fits [40].

**Table 2:** Estimated parameter values and their 95% confidence intervals as well as the maximum log-likelihoods (L) and corrected Akaike Information Criterion (AIC_C_) for each co-limitation model as presented in Fig. 6.

Parameter	Model 1a	Model 1b	Model 2a	Model 2b
K_p_ (nM)	1.09 [0.77, 1.49]	1.46 [1.06, 1.85]	0.70	N/A (0.88)
K_c_ (µM)	0.38 [0.21, 0.57]	1.43 [1.00, 2.16]	N/A (1.51)	0.77[0.36–1.57]
*α*_Cmax_ (2a)	N/A	N/A	0.039 [−0.987, 0.193]	
*α*_Pmax_ (2b)				0.075 [0.056, 0.109]
μ_max_ (h^−1^)	0.073 [0.063, 0.083]	0.076 [0.065, 0.092]	0.059 [0.0432, 0.0588]	0.066 [0.057, 0.078]
L	128	126	102	126
AIC_c_	−250	−245	−198	−246

Model 1a: Independent co-limitation, multiplicative form; model 1b: Independent co-limitation, minimum form (Liebig's Law); model 2a: Dependent co-limitation (CO_2_ uptake depends on P-limitation), and; model 2b: Dependent co-limitation (P uptake depends on CO_2_-limitation). N/A = not applicable (K is calculated from *μ*_max_ *α*_max_^−1^), in model 2a model no confidence interval could be estimated for K_P_. The L and AIC_C_ were corrected for small sample size [40].

The models 2a, and 2b (Fig. 6C,D; Table 2) assume a dependent colimitation. In the model 2a, CO_2_ uptake depends on the P-limitation, resulting in an angular shape of the surface that shows an even stronger effect of P concentration on the growth rate (lowest K_p_, Table 2) and a weaker effect of CO_2_ concentration. Especially the data points at high CO_2_ concentration were badly fitted by the model. Given that the fitting was highly sensitive to starting values and that we found a difference in AIC_c_ compared to the fit of model 1a of ∼50, we conclude that model 2a does not reflect the underlying mechanisms of colimitation. In contrast, if we assume that P uptake depends on CO_2_-limitation (model 2b) we get a fit to the data similar to that obtained with the model 1b (Fig. 6D). There are no strong differences between the goodness of fit of model 1b and 2b as the AIC_c_ is similar (Table 2). The independent, multiplicative colimitation of model 1a resulted in the best fit, but the difference in AIC_c_ between model 1a and 2b is only ∼4.5. The residuals (observed-predicted) for the four models shown in Fig. 6 are visualised in Fig. S3.

## Discussion

When facing nutrient limiting conditions plants respond by increasing their ability for nutrient uptake. This can be established by increasing their maximum uptake rate (V_max_) and/or increasing their affinity for uptake (lower affinity constant, K_m_). V_max_ is positively related to the number of porters on the cytoplasmic membrane [8] whereas a change in K_m_ reflects the presence of a different porter type [23]. Because the response to single-nutrient or colimitation may differ, we analyzed the uptake kinetics of the green alga *Chlamydomonas acidophila* to different CO_2_ and P colimiting conditions and fitted the data to 4 different models describing colimitation.

We used semi-continuous cultures which implies that the strength of limitation decreases with increasing balanced growth rate. Moreover, uptake kinetics and cellular quota were directly converted into external nutrient concentrations that could then be related to balanced growth rate [41] and used to test different colimitation models. Because growth rates were used to calculate the external nutrient concentrations, the modeling results were considered carefully and conclusions were based in concert with the contour plots that show only measured kinetic characteristics. Enrichment experiments had revealed that growth in all cultures was colimited by CO_2_ and P, since the growth rates of both high and low CO_2_ acclimated cells were enhanced by increased P concentration (3.8–fold) but even more when both CO_2_ and P were supplemented (4.8–fold; [39].

### Single vs. multiple nutrient limitation

Cell homeostasis of balanced nutrient content is of the greatest importance for a proper functioning of enzymes and proteins, and consequently nutrient uptake and excretion rates are regulated to balance nutrient ratios within a certain range [15], [16]. Thus, survival and growth critically depend on an increase of the uptake capacity of *all* nutrients available in sub-optimal concentrations. This was reflected in the uptake response of colimited *C. acidophila* that had a high V_max,C_ when the cellular C content was low, and a high V_max,P_ when the cellular P content was low (Fig. 5). V_max_ was strongly related to Q_p_ (Fig. 3). Cell homeostasis therefore determined the limitation status of the cell, and consequently its nutrient uptake kinetics. The relationships of V_max,P_ and V_max,C_ versus Q_p_ for both high and low CO_2_ cells support previous observations that all cells were colimited for CO_2_ and P [39] and that acclimation to nutrient limiting conditions resulted mainly in changes in V_max_ (as exemplified in Fig. 1B; [31]). High CO_2_ cells had a lower cellular P content and were more severely P-limited whereas low CO_2_ cells had the lower cellular C content and were thus more severely CO_2_-limited. In addition, high CO_2_ cells possibly contained more C as a result of luxury accumulation caused by high CO_2_ concentrations, or their C content was increased by cellular accumulation of photosynthate products resulting from P-limitation (mainly lipids; [42]). The fact that V_max,P_ increases with increasing P-limitation has been amply demonstrated by a large number of observations in single nutrient, P-limited algal species [9]–[11], however, our colimitation results contrast with many single-nutrient studies in which V_max,C_ decreased with increasing CO_2_-limitation (e.g. [12], [43]). Thus, under colimiting conditions, CO_2_ and P uptake interact and single nutrient limitation studies cannot predict the cell response adequately. A similar conclusion was recently obtained in a cyanobacterium grown under N, Fe and N/Fe colimited conditions [44].

In P-replete cells of *C. acidophila* V_max,C_ was higher in high CO_2_ than in low CO_2_ conditions [12]. The same pattern was observed in many other P-replete algal species, which is explained by enhanced growth rates at high CO_2_ [14], [43]. If growth is not limited by either nutrient supply or light, high CO_2_ concentrations stimulate photosynthesis and consequently growth. In our experiments uptake and growth response to changing CO_2_ conditions was uncoupled in P-limited cells, revealed by the lower V_max,C_ in high CO_2_ P-limited cells that still have a higher maximum growth rate [31] than the low CO_2_ P-limited cells.

One intriguing result of our study is the colimitation for CO_2_ and P in cells growing at high CO_2_. The CO_2_ concentration measured in the CO_2_ aerated, algae-containing vessels was 330 µM independent of whether cultures contained 7.8 10^5^ or 1.5 10^6^ cells ml^−1^, nonetheless, calculations of external CO_2_ concentrations in the medium were a 100-fold lower (i.e 3 µM). A possible explanation is the presence of a diffusion barrier around the cells [45] despite the fact that *C. acidophila* is an active swimming flagellate with a strong chemotactic response to CO_2_ (pers. obs. and see [46] for *Chlamydomonas moewussii*).

The presence of such diffusion barrier can be inferred more clearly by looking closer at the difference between V_max,C_ and V_max,P_ and also at the definition of V_max_. V_max_ is a function of two parameters [8]: The number of porters divided by the handling time for nutrient uptake. High CO_2_ acclimated cells had the highest V_max,P_ showing they were under stringent P-limitation. Under P-limitation, cells excrete superfluously produced sugars from photosynthesis and cells producing a polysaccharide mucous layer will extend such layer under severe P-depletion [47], thus surrounding the cell with a diffusion barrier for nutrient uptake that increases handling time. Indeed, high CO_2_, P-limited cultures of *C. acidophila* had higher concentrations of dissolved organic substances than low CO_2_ cultures suggesting more (poly)saccharide excretion [31]. Presumably, this affects P uptake much less than C-uptake as V_max,P_ did not decline in the presence of a thick mucous layer in two other green algal species (mucilage twice cell radius; [48]), whereas CO_2_-uptake is considered seriously hampered by such diffusion barrier [49], [50]. This implies that high CO_2_, P-limited cells had to cope with increased handling time for CO_2_ uptake, which dampened the increase in V_max,C_ and the measured values of V_max,C_ underestimate the number of CO_2_-uptake porters actually present. Because low CO_2_ cells were also P-limited, a (lesser) diffusion barrier for CO_2_ uptake may likewise be present in these cells.

The low K_m,C_ in the high CO_2_, P-limited cells at low growth rate (Fig. 2b) also indicates that the CO_2_ concentration in direct vicinity of the cell was limiting and supports the presence of a diffusion barrier around the cells. This layer presumably increased in size with decreasing growth rate. The impact of a mucilage layer possibly explains the difference in response between a single nutrient limitation and colimitation in CO_2_-uptake kinetics, because P-replete cells will have a small or no diffusion barrier around the cells.

### Independent colimitation

The fact that model 1a delivered the best fit suggests that an independent, multiplicative colimitation for CO_2_ and P in *C. acidophila* is the best explanation. Liebig's Law of the minimum (model 1b), another form of independent colimitation, showed a worse fit, as did the two dependent colimitation models. But, beyond differences in goodness of fit, there are other arguments that support an independent colimitation. For instance, a basic assumption of Liebig's Law (model 1b) does not really apply, because the instantaneous maximum growth in a nutrient enrichment experiment was obtained when *both* CO_2_ and P were added [39]. The model 1b nevertheless fits reasonably well because one limitation (P) is considerably stronger than the other (CO_2_), which was confirmed by single-nutrient models (Fig. S1).

Regarding the dependent colimitation models (2a,b), their basic assumption does not agree with the results. The models assume changes in K_m_ over the range of nutrient limitation that did not occur in the experiments (see below for further discussion). The data show that, irrespective of the other nutrient limitation, the cells responded to a low nutrient content by increasing their V_max_ for that nutrient. On a cellular level this presumably resulted in a trade-off in space for porters of either nutrient on the cytoplasm membrane (see below). The contour plots support the conclusions from the model fittings as they reveal a trade-off in V_max_ for both limiting nutrients (Fig. 5A,B).

### Dependent colimitation

We expected to find that CO_2_-acquisition depends on P-limitation [25], [31], [51], since the realization of a low K_m,C_ and a CCM [25] are both active processes hampered by insufficient ATP during P-limitation [28]. Under P-replete, low CO_2_ conditions, *C. acidophila* had both a low K_m,C_ and a CCM [12], [29]. However, the K_m,C_ was also unexpectedly low in stringent P-limited cells (Figs. 2B, 3B). Thus, our results support those of Kozlowska et al. [51], who showed a lower K_m,C_ in P-limited than in P-replete cells of *Chlorella vulgaris*. Possibly, *Chlorella vulgaris* was also colimited by P and CO_2_ in their study as cell densities were high, whereas in the study of Beardall et al. (K_m,C_ higher in P-limited than P-replete low CO_2_ cells) the cell density of *Chlorella emersonii* was low [25]. Of course other explanations such as the presence of entirely different adaptation mechanisms in the different species of *Chlorella* are also possible. Nonetheless, the poor fit of our data to model 2a (which reflects these assumptions) clearly results in a rejection of a dependent colimitation in which CO_2_ acquisition depends on P-limitation in *C. acidophila*.

Model 2b (P-uptake depended on CO_2_ limitation) provided a reasonable fit to the data and it supports our earlier finding that high CO_2_, P-depleted cells of *C. acidophila* had a higher P uptake ability (i.e. realized a lower external P concentration) than low CO_2_ cultures [30]. However, we think that the model fits for the wrong reason, as the presence of a dependent colimitation would imply that the K_m,P_ is higher in the low CO_2_ cells (i.e. more stringent CO_2_-limited), whereas we find the direct opposite (related to growth rate) or no difference (related to Q_p_). The relative good fit of model 2b must therefore be a result of the enhanced growth capacity for P in the high CO_2_ cells (Table S1) compared to low CO_2_ cells. Again, the contour plots support the conclusions from the model fittings, as changes in K_m_ and affinity for both limiting nutrients did not follow the expected changes based on dependent colimitation models (Fig. 5).

### Space limitation

Our data show a trade-off between V_max,P_ and V_max,C_ (Fig. 4): when P uptake ability (V_max,P_) was high, that for CO_2_ (V_max,C_) was low and *vice versa*. Nutrient uptake modeling revealed that V_max_ is directly and positively related to the number of porters, although increased handling time can dampen this relation [8]. Also experimentally, in higher plant cell cultures (guard cells of *Solanum tuberosum*, *Nicotiana tabacum* and *Vicia faba*) a positive relation was found between the K^+^-uptake porter density and K^+^ transport capacity [52]. Therefore, V_max_ can be used as an indicator for the number of active porters. Accordingly, we conclude that the number of porters was related to the cellular content of the limiting nutrient, but also to its external concentration. This relation not only holds for micro-organisms or plant cell cultures but also for higher plants as, for example, the density of stomata (porter for gas) declined linearly with air CO_2_ concentration [53], although there is much debate at this point [54].

An algal cell requires many different nutrients for growth, and all need transportation through the cytoplasm membrane by (often) nutrient-specific porters. Calculations on the number of nitrate porters in a hypothetical algal cell revealed that 8.5% of the cell surface may be covered by just one type of porter [8], [23]. Within the constraint of an overall fixed number of porters, a cell can only increase the density of porters for a specific limiting nutrient at the expense of others [23]. Under colimiting conditions, it seems plausible that a space limitation for porters on the membrane results in a trade-off in the investment for porters for those limiting nutrients [24], for which we provide the first empirical evidence.

At the cellular level, the trade-off in V_max_ can be interpreted as a kind of dependent colimitation: The space freed by a decrease in number of porters for one nutrient is used for an increase of porters of another one. In contrast, current dependent colimitation models assume that the concentration of one limiting nutrient has an effect on the K_m_ for the acquisition of another one and not on V_max_. This requires a different set of models where adaptation in V_max_ is considered (possibly starting from [55]).

Increasing evidence reveals that phytoplankton in marine and freshwater ecosystems and plants in general are colimited in their growth, and our study enhances the understanding of phytoplankton growth response and physiological adaptation under a colimitation for CO_2_ and P. In conclusion, cell nutrient homeostasis regulated nutrient acquisition in *C. acidophila*, and the most plausible mechanism was a multiplicative, independent colimitation. Given the space constraints on the cytoplasm membrane a trade-off in the number of porters for the uptake of different nutrients seems plausible under colimiting conditions. Responses to colimitation cannot be predicted from those to single nutrient limitation and therefore experiments on colimited plants are required to properly predict growth responses to a complex and changing natural environment. Our conclusions may also apply for other nutrients such as K and P [30], Si and P [56], N and CO_2_ [27], [57]–[59] in algae and in higher plants [52], [53], [60].

## Supporting Information

Figure S1Balanced growth rates (h^−1^) fitted to external CO_2_ concentration (A: Sc, in µM) and P concentration (B: Sp, in nM) using a single nutrient Monod model. Prior to testing colimitation models, we fitted single-nutrient models to check if one nutrient alone can satisfactorily explain the growth response of *C. acidophila*. For this, we used a standard Monod function with the external concentration of either carbon or phosphorous as predictors, as:
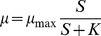
where *S* represents either carbon or phosphorous concentrations in the medium. The ability of carbon concentration to explain growth response was quite low, compared with other models, which is coherent with the high dispersion evident in the data (Fig. S1a). Additionally, the maximum growth rate predicted by this model was much lower than all the others. On the other hand, phosphorous had a much better predictive ability (Fig. S1b), which is also consistent with the stronger effect of phosphorous detected in the colimitation models. Still, the model with phosphorous alone had a worse fit than most of the colimitation models, suggesting again that growth is better described based on a multiple-nutrient colimitation. The modelling of μ to CO_2_ resulted in an estimation and 95% confidence interval of *μ_max_* of 0.036 [0.029 0.043], *K_C_* of 0.97 [0.42 1.74], Log-likelihood of 75.5, and corrected Akaike Information Criterion (AIC_C_) of −148.3. The modelling of μ to P resulted in an estimation and 95% confidence interval of *μ_max_* of 0.059 [0.054 0.065], *K_P_* of 1.08 [0.9 1.38], Log-likelihood of 105.8, and AIC_C_ of −208.9.(TIF)

Figure S2 Cellular carbon (A, in pmol C cell^−1^) and cellular phosphorus (B, in fmol P cell^−1^) content of *Chlamydomonas acidophila* in relation to balanced growth rate (d^−1^) of high CO_2_ (+CO_2_) and low CO_2_ (−CO_2_) P-limited cultures. Mean ± SE of 3 measurements. CO_2_ concentration had a significant effect on the cellular C content (ANCOVA, df = 1,27, F = 5.9, p<0.05) and cellular P content (ANCOVA, df = 1,27, F = 12.5, p<0.01) when the effect of growth rate is accounted for.
(TIF)

Figure S3 Residuals (observed-predicted) for the four models shown in Fig. 6 in the main text. A) model 1a; B) model 1b; C) model 2a; and D) model 2b.
(TIF)

Table S1 Calculated growth capacity, i.e. V_max_/cellular nutrient content (d^−1^), of *Chlamydomonas acidophila* in relation to balanced growth rate (d^−1^) of high CO_2_ (+CO_2_) and low CO_2_ (−CO_2_) P-limited cultures at pH 2.7. The following assumptions were made: 1) The maximum uptake rate is for 100% converted into growth during the 16 h light period per day, and 2) 1 mol O_2_ is released when 1 mol CO_2_ is fixed (required to calculate V_max,C_). Calculated growth capacity based on maximum CO_2_ uptake rates revealed a higher capacity in the low CO_2_ cells than needed to maintain balanced growth rate, whereas capacity equaled balanced growth rate in high CO_2_ cells. Calculated growth capacity based on maximum P uptake rate were >100-fold higher than balanced growth rates. Such overcapacity has been found more often in P-limited algal cultures [10], [62]. In addition, growth capacity was higher in the high CO_2_ than in the low CO_2_ cells.
(DOC)
