# Supplementary figures and images for: Independent Colimitation for Carbon Dioxide and Inorganic Phosphorus
Source: PLoS One. 2011 Dec 1;6(12):e28219. doi: 10.1371/journal.pone.0028219 (PMC3228739; doi:10.1371/journal.pone.0028219)

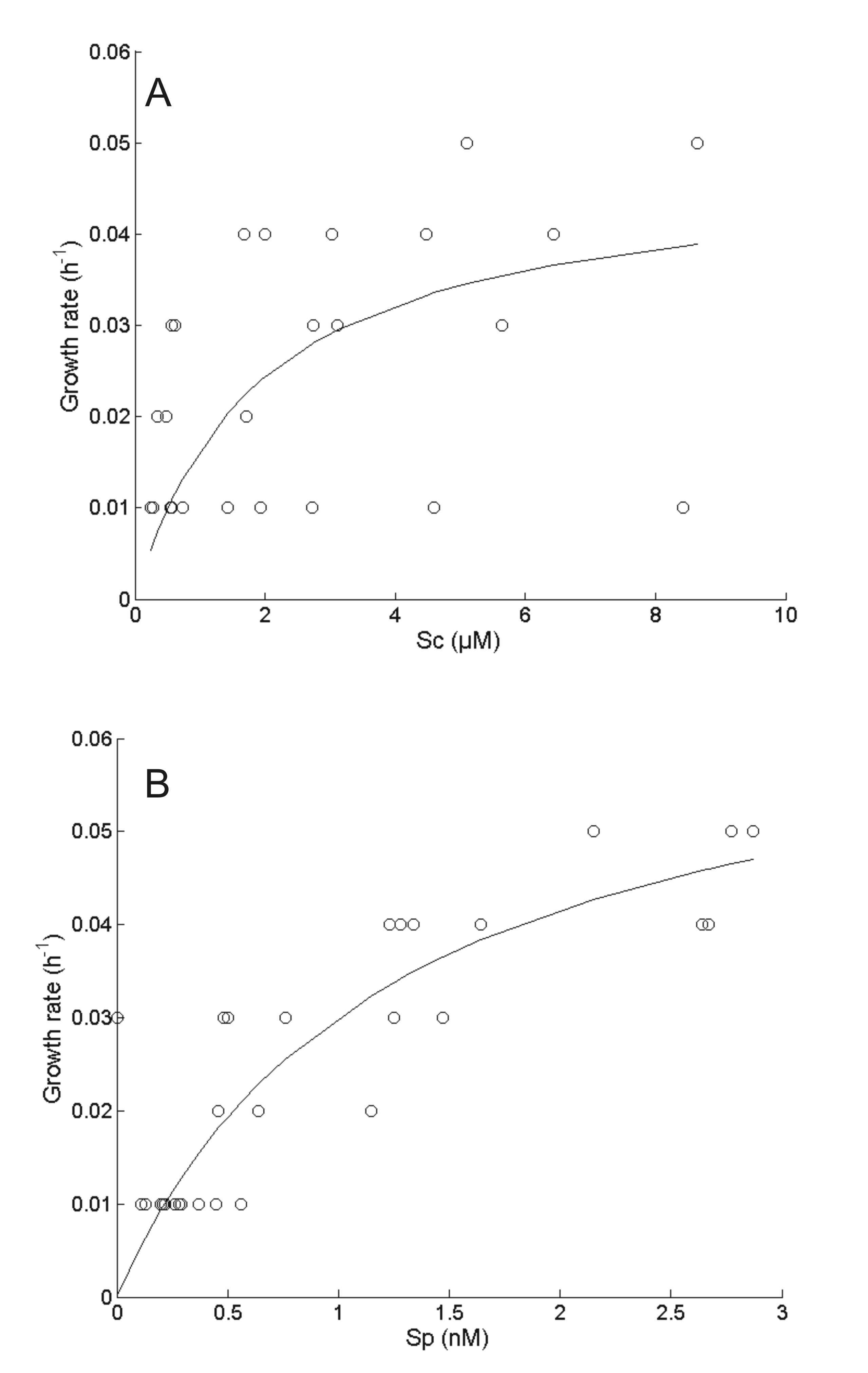

Supplement: Figure S1 — Balanced growth rates (h−1) fitted to external CO2 concentration (A: Sc, in µM) and P concentration (B: Sp, in nM) using a single nutrient Monod model. Prior to testing colimitation models, we fitted single-nutrient models to check if one nutrient alone can satisfactorily explain the growth response of C. acidophila. For this, we used a standard Monod function with the external concentration of either carbon or phosphorous as predictors, as:where S represents either carbon or phosphorous concentrations in the medium. The ability of carbon concentration to explain growth response was quite low, compared with other models, which is coherent with the high dispersion evident in the data (Fig. S1a). Additionally, the maximum growth rate predicted by this model was much lower than all the others. On the other hand, phosphorous had a much better predictive ability (Fig. S1b), which is also consistent with the stronger effect of phosphorous detected in the colimitation models. Still, the model with phosphorous alone had a worse fit than most of the colimitation models, suggesting again that growth is better described based on a multiple-nutrient colimitation. The modelling of μ to CO2 resulted in an estimation and 95% confidence interval of μmax of 0.036 [0.029 0.043], KC of 0.97 [0.42 1.74], Log-likelihood of 75.5, and corrected Akaike Information Criterion (AICC) of −148.3. The modelling of μ to P resulted in an estimation and 95% confidence interval of μmax of 0.059 [0.054 0.065], KP of 1.08 [0.9 1.38], Log-likelihood of 105.8, and AICC of −208.9. (TIF) [file pone.0028219.s001.tif]

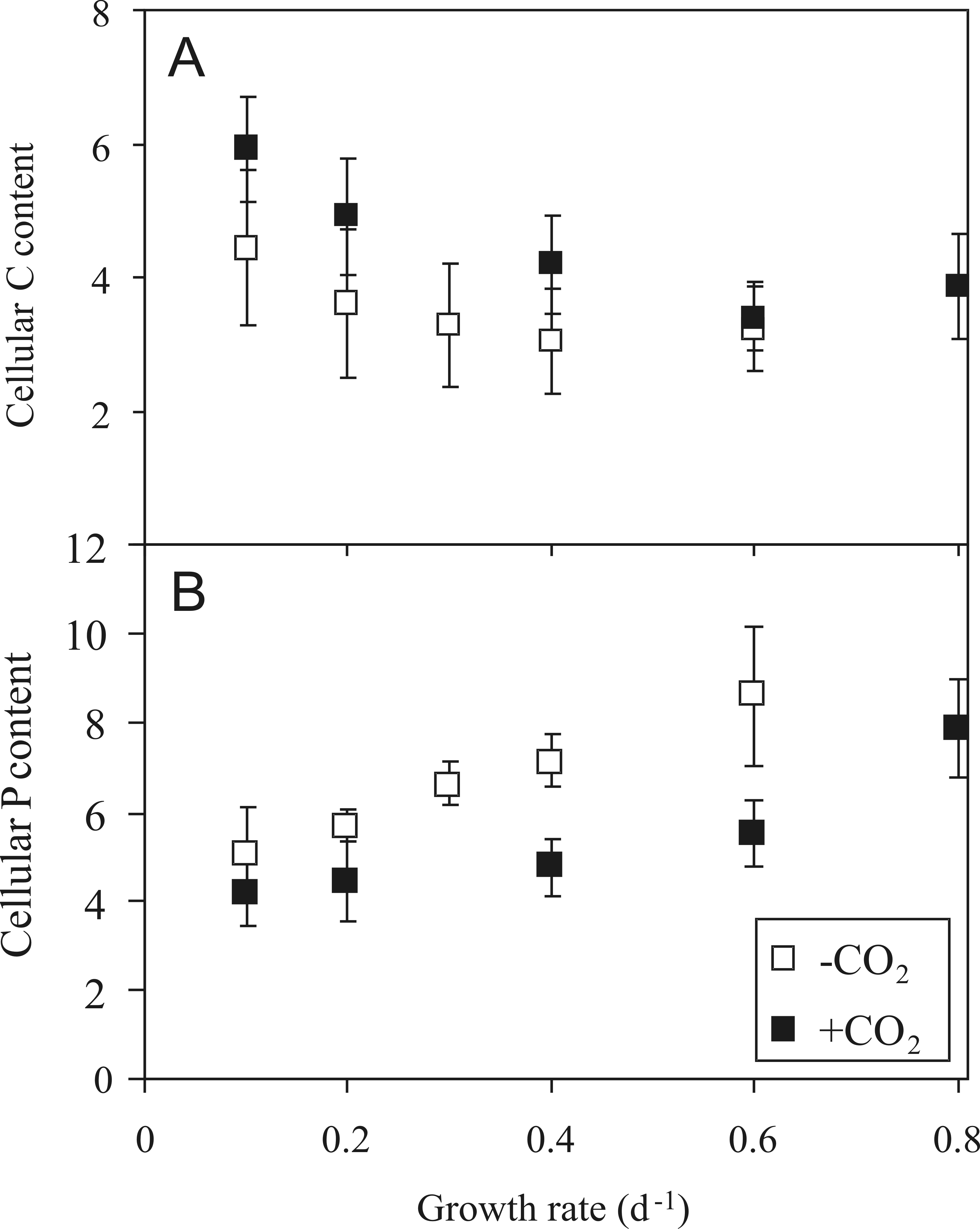

Supplement: Figure S2 — Cellular carbon (A, in pmol C cell−1) and cellular phosphorus (B, in fmol P cell−1) content of Chlamydomonas acidophila in relation to balanced growth rate (d−1) of high CO2 (+CO2) and low CO2 (−CO2) P-limited cultures. Mean ± SE of 3 measurements. CO2 concentration had a significant effect on the cellular C content (ANCOVA, df = 1,27, F = 5.9, p<0.05) and cellular P content (ANCOVA, df = 1,27, F = 12.5, p<0.01) when the effect of growth rate is accounted for. (TIF) [file pone.0028219.s002.tif]

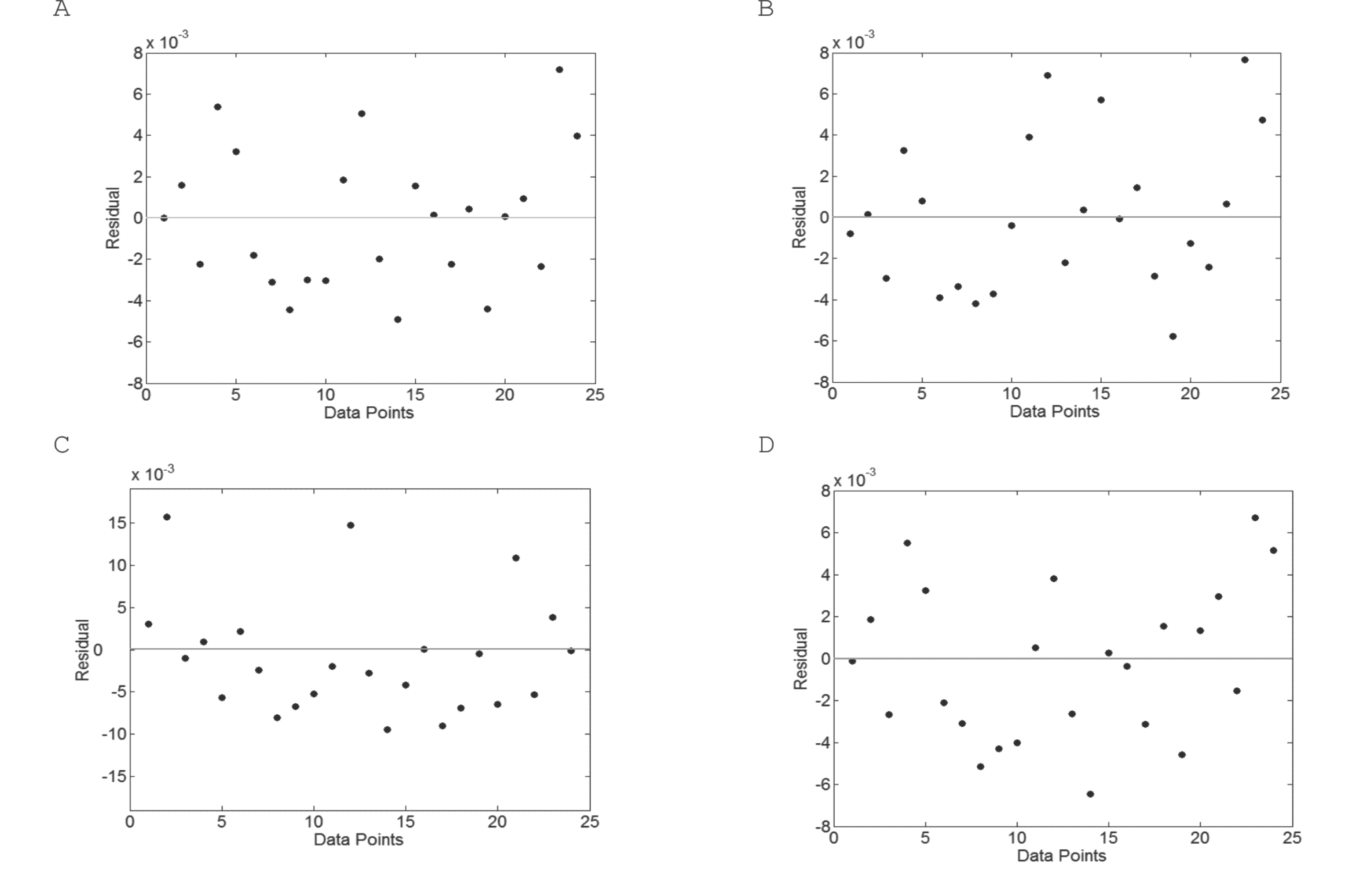

Supplement: Figure S3 — Residuals (observed-predicted) for the four models shown in Fig. 6 in the main text. A) model 1a; B) model 1b; C) model 2a; and D) model 2b. (TIF) [file pone.0028219.s003.tif]
