# Supplementary material for: Independent Colimitation for Carbon Dioxide and Inorganic Phosphorus
Source: PLoS One. 2011 Dec 1;6(12):e28219. doi: 10.1371/journal.pone.0028219 (PMC3228739; doi:10.1371/journal.pone.0028219)
Supplement: Table S1 — Calculated growth capacity, i.e. Vmax/cellular nutrient content (d−1), of Chlamydomonas acidophila in relation to balanced growth rate (d−1) of high CO2 (+CO2) and low CO2 (−CO2) P-limited cultures at pH 2.7. The following assumptions were made: 1) The maximum uptake rate is for 100% converted into growth during the 16 h light period per day, and 2) 1 mol O2 is released when 1 mol CO2 is fixed (required to calculate Vmax,C). Calculated growth capacity based on maximum CO2 uptake rates revealed a higher capacity in the low CO2 cells than needed to maintain balanced growth rate, whereas capacity equaled balanced growth rate in high CO2 cells. Calculated growth capacity based on maximum P uptake rate were >100-fold higher than balanced growth rates. Such overcapacity has been found more often in P-limited algal cultures [10], [62]. In addition, growth capacity was higher in the high CO2 than in the low CO2 cells. (DOC) [file pone.0028219.s004.doc]

| Balanced | Calculated growth rate based on V_max,C_ | | Calculated growth rate based on V_max,P_ | |
| --- | --- | --- | --- | --- |
| growth rate | +CO_2_ | -CO_2_ | +CO_2_ | -CO_2_ |
| 0.1 | 0.08 | 0.44 | 314 | 233 |
| 0.2 | 0.24 | 0.74 | 328 | 247 |
| 0.3 |  | 1.00 |  | 184 |
| 0.4 | 0.45 | 1.00 | 335 | 174 |
| 0.6 | 0.80 | 1.03 | 250 | 100 |
| 0.8 | 0.78 |  | 165 |  |
